# Presence of Institutional Guidance on Research-Related Transportation Could Help Reduce Barriers to and Disparities in Research Engagement

**DOI:** 10.1017/cts.2025.10052

**Published:** 2025-05-26

**Authors:** Nicholas Prestayko, Pilar N. Ossorio, Abbey Fisher, Nikhil Menon, Danielle Symons Downs, Lea G. Yerby, Aleksandra E. Zgierska

**Affiliations:** 1 Family and Community Medicine, The Pennsylvania State University College of Medicine, Hershey, USA; 2 Clinical and Translational Science Institute, The Pennsylvania State University College of Medicine, Hershey, USA; 3 University of Wisconsin-Madison Law School, Morgridge Institute for Research, Madison, USA; 4 School of Science, Engineering, and Technology, The Pennsylvania State University Harrisburg, Middletown, USA; 5 Kinesiology, The Pennsylvania State University College of Health and Human Development, University Park, USA; 6 The University of Alabama College of Community Health Sciences, Tuscaloosa, USA

**Keywords:** Transportation, recruitment, retention, research participation, research engagement

## Abstract

**Introduction:**

Lack of reliable, affordable transportation is a common barrier to clinical research participation, potentially contributing to health disparities. Insufficient and/or nonexistent institutional policies on research-related transportation make it challenging for research teams to effectively overcome transportation barriers and promote research participation among people from disadvantaged backgrounds. This study’s goal was to review research-related transportation policies across clinical research-involved institutions and propose recommendations for what such policies should address to help promote research engagement among diverse, representative populations.

**Methods:**

We surveyed 28 recruitment sites, members of the National Institutes of Health-funded Healthy Brain and Child Development Consortium, poised to recruit over 7000 families, and completed an online search for each site’s policies relevant to research-related transportation (i.e., transportation of participants or research staff travel to/from research activities). We identified, reviewed, and thematically described content of the relevant policies and developed summary recommendations for institutional guidance components.

**Results:**

We identified seven policies (from five sites) on research-related transportation; four provided guidance on research-related transportation services; two on reimbursement; and one on when research staff transports participants. The online search identified publicly available business travel policies for 22 sites. No policy addressed research staff travel specifically for “study business” or research personnel transporting children for research purposes.

**Conclusions:**

Few institutions involved in clinical research have policies guiding research-related transportation. Such policies, if adopted, could help support research-related transportation and, thus, participation of individuals from disadvantaged backgrounds, increasing generalizability of research results and contributing toward reducing social and health disparities.

## Introduction

Transportation barriers are a critical issue in clinical research, particularly for underserved and underrepresented populations. Equitable participation is essential for generating generalizable knowledge that improves health outcomes for all [1]. Historically, disadvantaged and marginalized groups—including minoritized populations, individuals with disabilities, and those in rural areas—have been underrepresented in clinical research, leading to findings that may not adequately reflect diverse health needs [2–8]. While studies show these groups are often willing to participate [9], structural barriers, including transportation limitations, significantly impact their ability to do so. Transportation challenges particularly affect socioeconomically disadvantaged and racially minoritized groups [10]. Addressing logistical challenges is crucial for increasing research engagement across broad populations. Research results may lack broad applicability if study samples do not account for genetic, biological, cultural, behavioral, or environmental differences, which affect disease presentation, progression, and treatment responses [2–5].

Travel to and from study sites is a key deterrent to research participation [11–14]. The high costs associated with transportation can explain why this factor is a major deterrent to accessing clinical studies [12]. Clinical study visits typically occur in academic centers, and participants often need to travel substantial distances, even in excess of 100 miles, to access research sites; this is costly, effortful, and time-consuming [15]. This issue is particularly relevant for the HEALthy Brain and Child Development (HBCD) study, a complex, multisite NIH-funded birth cohort study aiming to enroll over 7,000 child–parent dyads and retain them for nearly a decade [16]. The study’s success depends on sustained participation from a diverse population, yet the initial survey of HBCD sites identified transportation as a major obstacle, and many sites reported inadequate institutional guidance on managing transportation logistics effectively [17]. Without clear institutional policies, research teams struggle to ensure participant access while managing financial and legal risks that can be associated with research-related travel.

While some transportation barriers may be difficult to overcome, others could be mitigated through institutional policies and sufficient funding allocated during the planning and budget development process to this aspect. Comprehensive policies could reduce travel-related challenges, benefiting both research teams and diverse participants, particularly in long-term studies. Institutional guidance on study personnel and participant travel for research-related activities could help teams navigate ethical, safety, financial, and regulatory complexities. Without clear policies, research teams may face undue financial and legal responsibilities, discouraging community-based research efforts that could enhance participant recruitment and retention.

A key policy consideration is travel by study personnel to participants’ homes or communities. Study personnel who might need to travel, following the research protocols, can include research staff paid by the study (e.g., research assistants), students who receive school credit for working on a research project, or unpaid volunteers working on behalf of the research project or institution. Institutions should clarify whether individual versus institutional insurance covers costs related to travel incidents, such as accidents or roadside assistance [17]. Additionally, reimbursement policies for study team members’ research-related travel should be well-defined to prevent financial burdens on staff [17].

Another crucial issue involves study personnel or volunteers providing transportation to participants and their accompanying persons. Research teams must navigate transportation-related ethical and legal concerns that policies could help address. Safety concerns arise when transporting research participants, especially vulnerable persons such as children or individuals with certain health conditions [17]. Particularly relevant to many pediatric studies, including the HBCD research, transporting young children presents unique challenges as improper use of car seats, or lack thereof, poses safety and liability risks. Institutional policies should provide explicit guidance on whether research personnel can transport research participants and accompanying persons, including children; who is responsible for providing and installing car seats; how safety can be promoted; and how potential liability should be managed. Addressing such concerns in advance can prevent legal complications and help teams determine whether and how research personnel will be involved in participant transportation.

Understanding liability protections available at each study site is crucial, especially concerning potential accidents during research-related transportation. “Liability” refers to the legal responsibility to compensate a third party injured by one’s negligence. For example, if a study coordinator injures an infant participant while securing them in a car seat, who bears the cost stemming from this injury? Similarly, if research personnel are involved in a vehicle collision while traveling for study purposes, who is responsible for any resulting injuries or property damage? Research institutions can mitigate liability risks through various insurance policies, notably automobile insurance and general liability insurance (GLI). GLI covers claims arising when business operations or employees, acting within their scope of employment, wrongfully cause bodily injury or property damage to third parties. Many states provide GLI to shield their employees from liabilities incurred during the negligent performance of official duties. Additionally, public employees, such as those at state universities, may benefit from sovereign immunity, offering further liability protection.

Providing financial support for participant travel is another key factor in reducing transportation-related barriers. Institutions must determine whether travel costs will be covered upfront or reimbursed later. Some participants face financial constraints, including wage losses and other expenses related to clinical research participation [15,18], that may prevent them from attending study visits [12]. Providing stipends in advance, rather than relying on retroactive reimbursement, could encourage participation. A structured approach to covering travel expenses, particularly for economically disadvantaged or rural participants, could enhance recruitment and retention [12,19,20]. A 2017 survey by the Michael J. Fox Foundation found that 95% of 49 clinical trial sites reported improving transportation infrastructure would enhance research engagement [12].

Some research teams address transportation barriers by contracting with existing services such as taxis, Uber, or Lyft [12]. While this approach can be effective, institutions need policies to guide these arrangements and ensure compliance with ethical and financial regulations [12]. Clear contracts and agreements with transportation providers can help avoid liability issues and unexpected costs [12]. Institutions should provide guidelines on selecting appropriate transportation services and establishing agreements that prioritize participant safety and convenience [12].

To reduce transportation barriers and improve engagement among diverse participants in the HBCD study, we reviewed institutional policies on research-related transportation within HBCD Consortium institutions to identify gaps in existing guidance. We also outlined recommendations for policies that support equitable access to research participation across different populations and age groups to ultimately enhance the generalizability of research findings.

## Materials and methods

### Design

This study resulted from discussions among personnel at HBCD recruitment sites [16] on how to best support research-related transportation. At the time of data collection, the consortium comprised 25 funded sites; however, because some of the sites included more than one research institution and recruitment location, there were 28 recruitment sites across the consortium. Information on institutional policies relevant to research-related transportation was collected using two methods: (1) an email-based brief survey of the recruitment sites and (2) an online search for each site’s relevant policies. This study did not meet the criteria for human subject research and did not require a review by the Institutional Review Board.

### Procedures

#### Research-related transportation policies

The email sent to the research coordinators and principal investigators of 28 recruitment sites included the following statement: “Policies related to transportation for research purposes can include considerations regarding liability insurance and coverage (e.g., in case of a car accident) and details on what scenarios and options are allowed versus not permitted. Here are the examples of policies that your institution may have in place to guide the following: a) study personnel or volunteers traveling to participant home/community; b) study personnel or volunteers providing rides to study participants (and their accompanying persons); c) study covering the cost of participant travel (either upfront or reimbursing the cost after participant incurs it); d) study/institution “contracting” (making arrangements) with existing transportation services (taxi, Lyft, Uber, etc.); and e) guidance on car seats for young children. Please send the policies you have at your site. The initial emails were sent to all sites on May 31, 2023. A reminder email was sent on June 7, 2023 to 15 sites, which had not responded to the initial request. Email response collection was closed on July 6, 2023.

The comprehensive online search for research-related transportation policies was performed (NP, PNO) for each site using the Google search engine. The following phrase was used to search (NP) for general transportation policies: “*Transportation policies for [SITE INSTITUTION’S NAME].*” Contents of the first two pages yielded by this search were reviewed to find any information about relevant institutional policies. A limit of two pages was selected after a preliminary search determined that relevant information was not found after the second page. This search was completed by NP twice, first in the fall of 2023 then repeated in the fall of 2024.

#### Business travel policies

Search for business travel-related policies, which could be relevant to study personnel-provided transportation for research purposes, was completed by PNO in the first 2 weeks of June 2024, then repeated in the last 2 weeks of June and again in the 3^rd^ week of September 2024, using several phrases.

For policies on reimbursing the use of private vehicles for official business, the phrase was: “*[SITE INSTITUTION’S NAME] reimbursement for use of personal vehicle for [SITE INSTITUTION’S NAME] business*” and “*[SITE INSTITUTION’S NAME] use of personal vehicle for business.*”

For policies on liability and insurance coverage, the phrases were “*[SITE INSTITUTION’S NAME] employee general liability coverage*;” “*[SITE INSTITUTION’S NAME] employee general liability insurance;*” and “*[SITE INSTITUTION’S NAME] insurance of personal vehicle used for business purpose,*” with the last search phrase repeated while substituting the terms “*automobile*” then “*car*” for “vehicle.” In addition, PNO also searched each institution’s website, without the institution’s name at the beginning, and examined each institution’s risk management department’s web pages (if the institution had such a department and/or the website was publicly accessible).

### Data collection

Information yielded by email responses and through the online search for each site’s policies was included if it described institutional policies relevant to research-related transportation, business travel or insurance, with “policy” defined as any relevant written guidance. Information from identified policies was abstracted and summarized.

### Analytical approach

#### Research-related transportation policies

Based on the prior research [17], and feedback from the HBCD consortium’s Workgroups and study teams across all sites, we identified five main domains of research-related transportation as important to be addressed by institutional policies: (1) *Research staff travel for “study business”* (e.g., to meet study participants, pick up study-related materials or specimens); (2) *Research staff transporting research participants* (with or without participant’s companions); (3) *Research staff transporting a child for research purposes* (child-participant or a child who accompanies the participant) *when the child requires a car seat;* (4) *Reimbursement of participants’ research-related travel costs* (e.g., upfront versus later reimbursement, types of reimbursement methods); and (5) *Guidance or arrangements regarding the use of existing transportation services* (e.g., ride-share, shuttle, taxi, fleet vehicle, public transportation vouchers, passes, or tickets, etc.) *for participants’ research-related travel*. We planned to add new domains should the review of the institutional policies yield new themes not captured by the above consensus-determined five domains.

#### Business travel policies

We first identified the HBCD sites with publicly accessible business travel policies, then reviewed and summarized the content of these policies that could be of relevance to research-related transportation.

Results of the search for institutional policies describing insurance and liability protections were reviewed, and categorized as addressing (or not) two broad types of insurance: (1) GLI coverage and (2) insurance coverage or other liability protection for the use of *personal* vehicles for business travel (which could be for research purposes). We focused on whether a research institution insures employees driving *personal* vehicles (as opposed to rental or institutional fleet vehicles) for business purposes because policies on personal vehicles might exhibit more variation across institutions and because researchers might have a more substantial liability risk when using a personal vehicle.

A site was deemed to offer protections against a risk if the institution’s website, formal written policy, or other legal document clearly stated that a risk was insured or that the institution and employees were immune from liability under state law. We coded “Yes” to liability protection if the institutional policy stated it. We coded “No” to liability protection if one of two conditions applied: (1) an institutional policy clearly stated that employees were not protected and we found no contrary information or (2) the institution had a risk management webpage discussing automobile insurance that addressed insurance for university-owned/leased vehicles, rented vehicles, student group/organization rentals, and international rentals, but not for the use of personal vehicles, and we found no other information indicating that the institution provided some protection for use of personal vehicles. We coded an institution’s liability protection as “Undetermined” if there was “insufficient information” to ascertain “Yes” or “No” status; for example, if relevant insurance or legal protections were mentioned, but details were so lacking that we could not ascertain whether the protection might extend to research team members (e.g., if the only mention of “general liability” on an institution’s website was a statement that the risk management department is responsible for handling claims of general liability, it was deemed as “insufficient information”).

## Results

### Research-related transportation policies

Of the 28 HBCD consortium recruitment sites contacted by email, 3 (10.7%) did not respond; 6 (21.4%) responded but did not provide any relevant information; 7 (25%) responded stating they did not have any relevant policies; and 12 (42.9%) provided some information on the policies they viewed as relevant. Review of policies emailed by these 12 sites revealed that only 4 policies (from 4/12 sites; 33%) contained information specific to research-related transportation; the remaining 8 (67%) submitted policies on employee business travel (*n* = 5 sites) or patient transportation for clinical visit purposes.

The online search yielded 3 additional policies on research-related transportation; two of them came from the sites already identified as having such policies via email communication. Therefore, the total number of policies on research-related transportation reached 7 (from 5 different sites). The identified seven policies did not yield information that would have justified the creation of an additional domain, beyond the existing five.

These policies provided some guidance pertaining to three of the five domains important to research-related transportation (Table 1): *research staff transporting research participants* (*n* = 1 policy); *reimbursement of participants’ research-related travel costs* (*n* = 2); and *the use of existing transportation services for participant research-related travel* (*n* = 4). None of the 7 policies covered the remaining two domains of interest: *transporting a child for research purposes when the child requires a car seat*; or *research staff travel for “study business.”*

**Table 1.** Institutional policies (*n* = 7) addressing the domains relevant to research-related transportation

Institutional guidance across five domains relevant to research-related transportation	Number of policies addressing a given domain	Excerpts from the existing policies
1. Research staff travel for “study business” (e.g., to meet study participants, pick up study-related materials or specimens).	0	No policy directly addressed research personnel travel for research-related responsibilities. However, some of the institutional business travel policies (see Table 2) could be applicable.
2. Research staff transporting research participants (with or without participant’s companions).	1	Site #1 [SM-1]: “Our department provides transportation for clients who are unable to drive to [Institution]. Are the client’s family members or friends allowed to accompany the clients in [Institution] vehicles? Parents, guardians, spouses, children and/or caregivers may, for various reasons, need to accompany patients or research clients to their appointments at [Institution]. It is acceptable for these people to accompany the patient/client in a [Institution] vehicle as space in the vehicle allows and as long as transportation does not require special equipment (i.e., an infant car seat).” This cited policy complies with this domain as long as the staff members transporting the participants (with or without the participant’s companions) are research staff employees.
3. Research staff transporting a child for research purposes (child–participant or a child who accompanies the participant) when the child requires a car seat.	0	No policy addressing this issue was found.
4. Reimbursement of participants’ research-related travel costs (e.g., upfront versus later reimbursement; types of reimbursement methods).	2	Site #2 [SM-2]: “All subject compensation (including stipends) and reimbursement must be described in the consent form. The consent form must outline the amount, method of payment (check, gift card, etc.) and timing of compensation and/or reimbursement for the IRB to consider the compensation plan.” “The [Institutional Review Board] IRB generally prefers payment to be made after each study visit. It is not appropriate to hold payments until all study visits (balloon payments) are complete. Further, participants who voluntarily dropout or are withdrawn by the investigator should receive compensation for any completed activities on a prorated basis.” Site #3 [SM-3]: “- Reloadable prepaid debit card - Payment by check (nonreimbursement) - Request a cash advance (when cash is needed to distribute to participants on-site) - Non-cash payments (i.e., gift certificates, T-shirts, etc.) […]” - Reimburse expenses incurred by participants (for expenses such as travel, parking, daycare) […].”
5. Guidance or arrangements regarding the use of existing transportation services (e.g., ride-share, shuttle, taxi, fleet vehicle, public transportation vouchers, passes, or tickets, etc.) for participants’ research-related travel.	4	Site #3 [SM-4]: “Our partnership with Lyft enables schools and departments to work with Lyft to set up custom transportation programs that support the needs of your group. Examples of how departments can utilize Lyft for official business: […] Patient or healthcare transportation to support research […].” Site #1 [SM-5]: “[Institution] has extended its Uber Health program from [Institution] to research operations across campus, which is in addition to traditional transportation offerings.” Site #4 [SM-6]: “[Institution] has partnered with Modivcare, a fully HIPAA compliant platform that gives healthcare professionals the power to order on-demand rides for patients without the headache of paperwork, faxing or tracking down patients.” Site #5 [SM-7]: “Participant Transportation Services for Non-Hospitalized Study Participants.” [Name of Transportation Service that the Institution] has a master agreement. To set up an account for a study, contact [staff member’s name].”

SM = Supplemental Material; the number following “SM” indicates a reference to a specific written institutional policy or guidance; the SM references can be found in the Supplemental Material document.

**Table 2.** Domains of institutional business travel policies (*n* = 22 sites) of potential relevance to research personnel, volunteers or independent contractors traveling for research-related business

Domain of business travel policy	Number of sites with business travel policies [references]
Driver requirements for anyone driving on behalf of the institution or leasing/renting vehicles from the institution, including all employees, volunteers, and independent contractors	**4** [SM: 8, 20, 40, 44]
Vehicle physical safety (maintenance, inspection, security, and safety) for university-owned vehicles, leased vehicles, rental vehicles, or personal vehicles used for business purposes	**8** [SM: 8, 20, 22, 25, 37, 41, 43, 44]
Accident/incident reporting for all drivers when traveling for business purposes	**3** [SM: 8, 20, 42]
Personal vehicles	**6** [SM: 8, 29, 34, 37, 41, 43]
Rental cars	**8** [SM: 12, 15, 17, 22, 25, 29, 42, 44]
Ride-share service policies for employees on business travel	**8** [SM: 12, 15, 17, 22, 25, 37, 43, 44]
Personal vehicle mileage reimbursement guidelines for employees	**9** [SM: 15, 17, 22, 25, 29, 31, 34, 37, 42]
Employee carpool/transportation programs	**5** [SM: 9, 11, 12, 13, 46]

SM = Supplemental Material; the number following “SM” indicates a reference to a specific written institutional policy or guidance; the SM-references can be found in the Supplemental Material document.

Although some of the institutional business travel policies could – at least partially – be applied to this latter domain (as described below), they did not explicitly address travel for research purposes.

### Business travel policies

By email communication, we identified five sites with business travel policies; these policies were also found through the online search. Overall, the online search identified 22/28 sites as having publicly accessible policies on business travel. Although these policies did not specifically address travel for “study business,” their review identified several areas of potential relevance to research-related transportation (Table 2). The most frequently addressed areas of business travel policies were: *Personal vehicle mileage reimbursement for employees* (*n* = 9 policies); *Ride-share service policies for employees* (*n* = 8); *Vehicle maintenance, inspection, security, insurance, and safety for institutionally-owned, leased or rental vehicles, or personal vehicles used for business purposes* (*n* = 8); *Rental cars* (*n* = 8); and *Personal vehicles* (*n* = 6). Less frequently addressed areas were: *Employee carpool/transportation programs* (*n* = 5); *Driver requirements* (*n* = 4); and *Accident/incident reporting* (*n* = 3).

Our search for institutional policies on liability protections identified 18/28 (64%) sites as having publicly accessible policies describing GLI protections likely applying to research team members who are institutional employees. All research institutions probably have GLI because it is a standard risk-management practice for businesses, but we failed to find relevant or sufficient information on the existence or scope of GLI for 10 sites (Table 3).

**Table 3.** Institutions providing insurance or other liability protection (*n* = 28 institutions)

Sites with:	Yes	No	Undetermined (insufficient information)
General liability insurance (GLI) for employees	18	0	10
Some insurance coverage or other liability protection for employees when using *personal* vehicle for business travel	17	2	9

Most researchers at public universities within our study sample likely have sovereign immunity from liability suits. Historically, U.S. states and their employees were immune from lawsuits by their citizens for injuries resulting from official actions. In the 20^th^ century, all states waived some of this immunity through constitutional amendments, legislation, judicial decisions, or combinations thereof [21]. Nonetheless, state university employees are generally immune from negligence liability when acting within their official duties, including research activities or related travel. Most HBCD personnel employed at state universities are covered by sovereign immunity. For instance, employees of the University of Maryland are immune from lawsuits for negligence committed within their scope of employment, even for auto accidents. However, the state of Maryland can be sued for an employee’s negligence. It is important to note that state immunity protections typically do not extend to individuals employed solely by private research institutes or private hospitals associated with public universities.

We found information on insurance for the use of *personal* vehicles for business purposes for 17/28 sites (61%) (Table 3). These policies had some important limitations. Eight sites’ policies described the institution’s auto insurance as secondary to the driver’s (or vehicle owner’s) personal insurance when a personal vehicle was used for business. Secondary insurance is meant to pay out only if liability for the accident is above the primary policy’s insurance limit; therefore, the secondary insurance provided by the research institution may not be obligated to pay if there is no primary insurance (i.e., if the research staff member driving a personal vehicle lacks personal auto insurance) [22]. Nine sites stated in their policies that when a privately owned vehicle is used for business purposes, the institution’s auto insurance policy only covers liability to third parties and does not cover personal injury to a researcher-employee or damage to the employee-driven personal vehicle. At the same time, 26/28 sites described insurance policies on liability for accidents for institutionally owned/leased vehicles (fleet vehicles) and/or rented vehicles.

## Discussion

Findings from this study illustrate the scarcity of institutional policies and other written guidance for researchers that explicitly address travel for research purposes, whether it involves study personnel driving to conduct “study business” or transporting research participants (and their companions) to facilitate their engagement in research. Among 28 surveyed sites, members of the NIH-funded HBCD consortium [16], we identified only seven policies, issued by five institutions, that specifically addressed some aspects of research-related transportation. By comparison, policies on business travel were more common, with 22 sites found to have online policies. All sites probably have such policies, but not all were publicly accessible. In addition, policies describing at least some aspects of liability protection and insurance for research personnel were found for approximately 60% of the sites. By comparison, almost all sites had easily available policies on the use of institutional/fleet or rental vehicles.

These findings are concerning because institutional policies and written guidance are crucial components of institutional-level support for research teams. Such policies educate researchers and influence how they address and strive to lessen transportation-related challenges for their study participants. Lack of appropriate policies can hinder recruitment and retention, especially among participants from populations and backgrounds that have historically been underrepresented in clinical research. In addition, this can negatively impact future funding potential for underperforming study sites (i.e., those with subpar recruitment or retention deliverables). Lack of explicit norms for supporting research participation, e.g., due to the lack of clear policies on research-related transportation, can (unintentionally) propagate prevailing inequities surrounding research engagement of marginalized groups.

Our results further highlight that transportation barriers to research participation may particularly impact studies involving children. None of the policies we identified addressed issues that may arise when transporting children, whether as participants or when accompanying an adult participant, especially when a car/booster seat is required. No policy described required training for the research staff to ensure child safety during transportation or what to do should the study team discover a lack of appropriate car/booster seat when the child needs one to travel. Institutional guidance is needed on the use of car/booster seats when transporting child participants, on driver requirements, and on liability considerations when transporting children.

This study’s findings also call attention to the challenges faced by research personnel when they need to travel for research-related business, particularly when transporting participants using employee-owned vehicles. Institutional policies should outline the requirements for the use of personal vehicles for research-related travel, emphasizing the importance of ensuring that the vehicles are safe (i.e., passed appropriate inspections per state requirements) and have current insurance coverage. Institutions should clearly explain whose insurance pays for any damages incurred while study staff are driving their personal vehicles for research purposes, including transporting participants and their companions.

Many institution’s policies discourage the use of personal vehicles for institutional business, but these policies are not designed for research. They are not well-suited to a context, in which study personnel involved in outreach to participants may need to drive frequently, at all hours, sometimes on short notice, and to numerous places not easily accessible by public transportation. The lack of research-related policies may create ambiguities in job roles, uncertainties regarding legal liabilities and financial responsibilities, even if the study personnel’s travel falls within a research protocol; this may affect motivation and morale among research personnel, with negative impacts on equitable participant recruitment, retention, and successful execution of clinical research [23,24].

This study also calls attention to the need for clearer policies regarding liability protection for students, contracted workers, and volunteers on study teams. Even when information about GLI and auto insurance is available to researchers, institutional policies often do not specify whether students who are receiving credit to conduct research (rather than serving as paid employees) are covered. In rare cases, students, contractors, or volunteers can be covered by an institution’s GLI or auto insurance when such people are acting on behalf of the institution, but often they are explicitly excluded from coverage, or their coverage is not addressed. Institutions should have policies and educational materials to help principal investigators navigate these complexities.

The results presented here document a need for institutions engaged in clinical research to have policies in place that support research-related transportation and remove barriers, including liability risks for staff conducting research in the field. The development and implementation of such policies is a critical step in creating equity in research. They are also needed for the research team members so they can understand up front the “roadmap” guiding research-related transportation and make informed decisions about, for example, using personal vehicles to drive research participants or complete home visits. Future studies will need to measure the effectiveness of research-related transportation policies on recruitment, retention, and research protocol adherence, particularly among historically underrepresented groups.

In summary, our findings inform the following recommendations for essential elements, across the salient domains relevant to research-related transportation, that we believe should be addressed by institutional policies or other written guidance, with the goal of supporting research teams and equitable research engagement of diverse participants (Table 4).

**Table 4.** Author recommendations for institutional guidance or policies on research-related transportation

Research-related transportation domains	Recommendations for institutional guidance
Research staff travel for “study-related business” (e.g., to meet study participants, pick up study-related materials or specimens).	Provide guidance for research personnel when they travel to meet participants or complete other study-related business: a. What are the requirements for a driver (e.g., presence of a valid driver’s license issued by a specific state?; auto insurance that covers occasional business activities?)? b. What are the allowed modes of transportation for study-related business travel? For example, can the research personnel use their personal vehicle? What are other options (e.g., rental or fleet vehicles)? c. If the use of personal vehicles for research-related business travel is allowed, what is the reimbursement rate (e.g., is it the Internal Revenue Service’s (IRS) standard rate per mile or another rate?) d. Specify details of liability insurance coverage for personnel/vehicles under each allowed transportation option when the personnel drive the vehicle (e.g., which insurance covers damages sustained in a motor-vehicle accident?) e. Specify details on roadside assistance under each allowed transportation option when the personnel drive the vehicle (e.g., who is covering the cost of roadside assistance and how to access it?).
	Provide guidance for research personnel on meeting study participants outside of research facility settings for study-related activities: a. Is meeting study participants for research-related activities allowed in public spaces or in participant homes? b. Outline considerations for ensuring research personnel safety as well as participant privacy and confidentiality. For example, what are the circumstances under which such a meeting needs to take place in a private room (there might be different considerations for when simply picking up a sample or materials from a study participant vs actively collecting data), and what types of data/specimens can be collected under each set of circumstances? When is it acceptable for one study team member to be present (e.g., to pick up materials from a study participant in a library) vs two or more research team members (e.g., to collect data in a public space or to complete a home visit)? Are there specific times of a day during which a meeting outside of the research facility is allowed (vs not)? For example, night-time meetings may not be as safe as those completed during daytime. c. Outline details of insurance coverage for injuries that may occur in the above contexts.
Research staff transporting research participants (with or without accompanying persons).	Provide guidance on whether research personnel are allowed to transport research participants (and/or their companions) for study-related activities, and under what circumstances (e.g., personal versus other type of vehicles?).
	Outline the driver requirements and provide guidance on the liability-related issues: who is liable (i.e., whose insurance covers the costs in case of an accident?) if a study team member transports research participants and/or their companions using personal vehicles versus other (e.g., rental or institution-owned fleet) vehicles.
	Outline the reimbursement rate for research staff when they use their personal vehicles to transport research participants for study-related activities (is it the IRS-defined standard mileage rate or other rate?). Outline safety considerations relevant to vehicles, drivers, and passengers. For example: Did the vehicle pass an annual maintenance/inspection; does it meet current safety standards (e.g., has functional air bags, seat belts)? Is the study participant allowed to sit in the front seat? What are the driver requirements (including zero tolerance policy for substance use (i.e., no driving under the influence of psychoactive substances)?
Research personnel transporting a child for research purposes (a child-participant or participant-accompanying child) when the child requires a car/booster seat.	Provide guidance on car/booster seats: Whose car/booster seat can be installed (i.e., study or institution-provided vs participant-owned seat?)? What is the approved process for car/booster seat installation (when a car seat is required) in the research personnel-operated vehicle (e.g., who should install the car / booster seat: staff versus caregiver?) Is there a required training for research personnel who transport children (i.e., completed child abuse training? Completed car/booster seat installation training? Completed CPR training?)
	Outline the driver requirements and liability considerations when research personnel transport a child (child-participant or child accompanying the participant) who requires a car/booster seat.
	Outline any needs regarding the minimum required number of adults in a vehicle when the child is transported and the need for a presence of caregiver and/or other approved adult.
Compensation for participants’ research-related travel cost.	Provide guidance on allowed (vs not allowed) methods of compensation (e.g., prepaid debit card, gift or gas cards, or cash) for research participant travel cost. To promote equity in research access, allow for multiple methods of compensation, including those not requiring a social security number or electronic account information from research participants.
	Provide guidance on allowed timing of compensation (upfront versus retrospective reimbursement) for research participant travel cost. To promote equity in research access and allow upfront participant compensation for travel-related costs.
Use of existing transportation services (e.g., ride-share, shuttle, taxi, fleet vehicle, etc.) for participants’ research-related travel.	Provide guidance on the use (allowed vs not) of the existing transportation services (e.g., ride-share, shuttle, taxi, fleet vehicle, etc.), including the use of public transportation vouchers, passes, tickets, etc., for participant’s travel. To reduce the burden on individual research teams, promote equity in research access among participants from diverse backgrounds, develop and put in place institutional agreements with the allowed “transportation services.”

IRS = Internal Revenue Service. CPR = Cardiopulmonary Resuscitation.

## Limitations

Although we attempted to systematically collect information on relevant policies, our communication with the HBCD study sites took place during a busy time of transitioning from the pilot to the main phase of the HBCD study; a strain on time and priorities could have led to a subpar response rate and incomplete results. Similarly, our online search could not have captured all relevant policies, with resultant underestimation of the presence of such policies. Because private research entities, such as private hospitals and research institutes, made sparse information public compared to public universities and hospitals, our study may be biased toward types of information disclosed by public entities. However, we believe it unlikely that private research institutions have policies that systematically or dramatically differ in content from those of public research institutions.

The prevailing sentiment from the research sites involved in clinical HBCD study, both in this and our prior survey [17], indicates that institutional policies specific and important to research-related transportation are uncommon. Anecdotally, research team members were not readily aware of the presence/absence of such guidance within their own site; it took time and effort to first search for such policies, before they were ready to respond to our email-based survey. Because the HBCD study sites comprise established centers engaged in federally sponsored clinical studies [16], it is likely our findings are representative across medical research institutions; however, it is possible sites outside of the HBCD Consortium may have policies guiding research-related transportation that are more extensive than those we identified.

## Conclusions

Research institutions engaged in clinical research, such as the HBCD study, have an opportunity to comprehensively support research teams and recruitment and retention of participants, particularly those from minoritized and disadvantaged backgrounds, by addressing a widespread lack of guidance on research-related transportation. The recommendations on institutional policies for research-related travel we outlined in this manuscript have the potential to fundamentally change how institutions, especially those with federally funded grants and contracts as stewards of public taxpayer dollars, are approaching this issue. With the guiding scientific principle of result generalizability, it is possible that research institutions, by implementing policies and recommendations on research-related transportation, can reduce barriers and encourage engagement among participants who have been underrepresented in research in the past.

## Supporting information

Prestayko et al. supplementary material Prestayko et al. supplementary material

## Supplementary material

The supplementary material for this article can be found at https://doi.org/10.1017/cts.2025.10052.
