# Supplementary material for: Presence of Institutional Guidance on Research-Related Transportation Could Help Reduce Barriers to and Disparities in Research Engagement
Source: J Clin Transl Sci. 2025 May 26;9(1):e128. doi: 10.1017/cts.2025.10052 (PMC12209963; doi:10.1017/cts.2025.10052)
Supplement: Prestayko et al. supplementary material [file S2059866125100526sup001.docx]

**Supplemental Material (SM)**

**SM References: Policies for research-related transportation**

1. The University of Alabama at Birmingham (UAB). Automobile Use FAQs. Published date unknown. Accessed on October 21, 2024 at: <https://www.uab.edu/riskmanagement/faqs/automobile-use-faqs>
2. Cedars-Sinai. Study Compensation: Payment and Reimbursement Guidance. Cedars Sinai, 2021. Obtained through personal communication, not available online.
3. University of Minnesota (UMN). Paying Human Participants Directly. UMN, 2024. Accessed on October 21, 2024 at: <https://policy.umn.edu/finance/humansubjects-proc01>
4. University of Minnesota (UMN). Custom Lyft Programs. UMN, 2024. Accessed on October 21, 2024 at: <https://pts.umn.edu/Transit/Transit-Services/University-Minnesota-Custom-Lyft-Programs>.
5. The University of Alabama at Birmingham (UAB). Uber Rides. Published date unknown. Accessed on October 21, 2024 at: <https://www.uab.edu/medicine/ctao/investigators/uber-rides>
6. Oregon Health and Science University (OHSU). Lyft for Patients. OHSU, 2023. Obtained through personal communication, not available online.
7. Johns Hopkins Institute for Clinical & Translational Research. Participant Transportation Services for Non-Hospitalized Study Participants. Published date unknown. Accessed on October 21, 2024 at: https://ictr.johnshopkins.edu/covid-research-center/hsr-process/implementation/participant-transport/

**SM References: Policies related to business transportation and general liability insurance**

1. Arkansas Children’s System (AC System). Fleet Management. AC System, 2021. Obtained through personal communication, not available online.
2. University of Arkansas for Medical Sciences (UAMS). UAMS Parking Operations: Easy Rider Program. UAMS, 2024. Accessed on October 21, 2024 at: <https://uams.edu/campusopsstaging/depts/po/easyrider>.
3. Arkansas Children’s Hospital, Bond Counsel Opinion, 2023. Accessed on October 16, 2024 at: <https://www.stephens.com/uploads/shared/transactions-pdf/Arkansas-Childrens-Hospital-OS.pdf>
4. Boston’s Children Hospital (BCH). Parking & Transportation. BCH, 2024. Accessed on October 21, 2024 at: <https://dme.childrenshospital.org/graduate-medical-education/benefits/parking-transportation/>.
5. Cedars-Sinai. Cedars-Sinai Staff Shuttle. Published date unknown. Accessed on October 21, 2024 at <https://www.cshsshuttle.com/>.
6. Emory University. Smart Commute. Transportation & Parking Services. Emory University, 2024. Accessed on October 21, 2024 at: <https://transportation.emory.edu/smart-commute>.
7. Emory University. Official University Policy, 2.90: Emory University Travel and Expense Policy. Emory University, 2018. Accessed on October 21, 2024 at: <https://inside.oxford.emory.edu/_includes/documents/sections/finance_admin/financial_services/Travel%20Policy.pdf>.
8. New York University (NYU). NYU Travel and Expense Policy. NYU, 2023. Accessed on October 21, 2024 at: <https://www.nyu.edu/about/policies-guidelines-compliance/policies-and-guidelines/business-expenses.html>.
9. New York University (NYU). Department of Insurance Programs. NYU, n.d. Accessed on October 21, 2024 at: <https://www.nyu.edu/about/leadership-university-administration/office-of-the-president/office-of-the-executivevicepresident/finance-and-budget/financial-operationsandtreasury/treasury-management/insurance-and-risk-management.html>
10. Northwestern (NW). Policies & Reimbursements. NW, 2024. Accessed on October 21, 2024 at <https://www.northwestern.edu/procurement/travel/policies.html>.
11. Northwestern (NW). Risk Management: Automobile Insurance. NW, 2024. Accessed on October 21, 2024 at: <https://www.northwestern.edu/risk/risk-insurance/university-insurance-programs/automobile-insurance.html>.
12. Northwestern (NW). Risk Management: Liability Insurance. NW, 2024. Accessed on October 21, 2024 at: <https://www.northwestern.edu/risk/risk-insurance/university-insurance-programs/liability-insurance.html>.
13. Oklahoma State University (OSU). Oklahoma State University Policy and Procedures. OSU, 2010. Accessed on October 21, 2024 at: <https://adminfinance.okstate.edu/site-files/documents/policies/transportation-services-motor-pool-and-motor-vehicle-service-station.pdf>.
14. Oklahoma State University (OSU). Administration and Finance FAQ. OSU, 2024. Accessed on October 16, 2024 at: <https://adminfinance.okstate.edu/rpm/risk-management/faq.html>
15. Penn State. TR02 Penn State Travel Policy. Penn State, 2020. Accessed on October 21, 2024 at: <https://policy.psu.edu/policies/tr02>.
16. Penn State, Office of Risk Management, Insurance. Penn State, 2024. Accessed on October 21, 2024 at: <https://fandb.psu.edu/office-risk-management/insurance>
17. Penn State, Business Services Policies BS20: University Vehicle Operations or Use. Penn State 2023, Accessed on October 21, 2024 at: <https://policy.psu.edu/policies/bs20#B>.
18. The University of Alabama (UA). Travel Policy and Procedures. UA, 2024. Accessed on October 21, 2024 at: <https://accountspayable.ua.edu/travel-policy/>.
19. The University of Alabama at Birmingham (UAB). The UAB/HSF Office of Risk Management User Guide. UAB, 2020. Accessed on October 21, 2024 at: <https://www.uab.edu/riskmanagement/images/2020_RISK_MANAGEMENT_MANUAL.pdf>.
20. The University of Alabama at Birmingham (UAB). You & UAB Handbook. UAB, 2020. Accessed on October 21, 2024 at: <https://www.uab.edu/humanresources/home/policies/uab-handbook>.

1. University of Alabama at Birmingham (UAB). UAB Financial Affairs, Travel Reimbursement Requests: UAB Employee. UAB, 2024. Accessed on October 21, 2024 at: <https://www.uab.edu/financialaffairs/images/documents/traveling/UAB_Employee_Travel_Reimbursement_Guide.pdf>.
2. The University of New Mexico (UNM). Administrative Policies and Procedures Manual - Policy 4030: Travel. UNM, 2019. Accessed on October 21, 2024 at: <https://policy.unm.edu/university-policies/4000/4030.html>.
3. The University of New Mexico (UNM). Risk Services: Liability Insurance. UNM, n.d. Accessed on October 21, 2024 at: <https://risk.unm.edu/property-and-liability-insurance/liability-insurance.html>.
4. University of California San Diego (UCSD). Travel Resources and Guidelines. UCSD, 2024. Accessed on October 21, 2024 at: <https://aabo.ucsd.edu/travel/travel.html>.
5. University of California San Diego (UCSD). Personal Vehicle Usage for UC San Diego Business. UCSD, 2023. Accessed on October 21, 2024 at: <https://blink.ucsd.edu/safety/risk/insurance/vehicle/personal.html>.
6. University of California San Diego (UCSD). Insurance: Automatic Coverage. UCSD, 2021. Accessed on October 21, 2024 at: <https://blink.ucsd.edu/safety/risk/insurance/about/automatic/index.html#General-liability>.
7. University of Maryland (UMD). University of Maryland Traveler’s Guide. UMD, 2021. Accessed on October 21, 2024 at: <https://www.dbs.umd.edu/travel/policy/umtravel/trav_guide.php>.
8. University of Maryland Extension, Maryland Tort Claims Act and Volunteers. UMD, 2021. Accessed on October 21, 2024 at: <https://extension.umd.edu/sites/extension.umd.edu/files/2021-12/Maryland%20Tort%20Claims%20Act%20and%20Volunteers.pdf>.
9. University of Maryland (UMD). Department of Environmental Safety, Sustainability, and Risk, Tort Claims. UMD, 2024. Accessed on October 21, 2024 at: <https://essr.umd.edu/about/risk-management/insurance/tort-claims>.
10. University of Minnesota (UMN). Traveling on University Business. UMN, 2021. Accessed on October 21, 2024 at: <https://policy.umn.edu/finance/travel>.
11. Minnesota Statutes 2023, 3.736 Tort Claims. Accessed on October 21, 2024, at: <https://www.revisor.mn.gov/statutes/cite/3.736/pdf>.
12. Universities of Wisconsin. System Administrative Policies, Vehicle Use and Driver Authorization. Universities of Wisconsin, 2022. Accessed on October 21, 2024 at: https://www.wisconsin.edu/uw-policies/uw-system-administrative-policies/vehicle-use-and-driver-authorization/#
13. University of Wisconsin-Madison (UWM). Driver Authorization. UWM, 2024. Accessed on October 21, 2024 at: <https://businessservices.wisc.edu/managing-risk/driver-authorization-and-insurance/driver-authorization/>.
14. University of Wisconsin-Madison (UWM). Automobile Insurance. UWM, 2024. Accessed on October 21, 2024 at: <https://businessservices.wisc.edu/managing-risk/driver-authorization-and-insurance/automobile-insurance/>.
15. University of Wisconsin-Madison (UWM). Vehicle Use/Rental: UW-3019. UWM, 2020. Accessed on October 21, 2024 at: <https://policy.wisc.edu/library/UW-3019>.
16. Vanderbilt University (VU).Travel and Business Expense Policy. VU, 2018. Accessed on October 21, 2024 at: <https://finance.vanderbilt.edu/policies/Travel_and_Business_Expense_Policy.pdf>.
17. Wake Forest University (WF). Enterprise Travel and Expense Policy. WF, 2022. Obtained through personal communication, not available online.
18. Wake Forest University (WF). Travel Administrative Procedure. WF, 2015. Accessed on October 16, 2024 at: <https://prod.wp.cdn.aws.wfu.edu/sites/141/2018/02/Travel-Procedure_01172017.pdf>
19. Washington University in St. Louis (WUSTL). Metro U-Pass. WUSTL, 2024. Accessed on October 21, 2024 at: <https://parking.wustl.edu/items/metro-upass/>.
20. Washington University in St. Louis (WUSTL). Financial Services: Property and Liability Insurance, WUSTL, 2024. Accessed on October 21, 2024 at: <https://financialservices.wustl.edu/wfin-topic/insurance-risk-management/notary-bonds/>.
21. Washington University in St. Louis (WUSTL). Financial Services: Vehicle Insurance. WUSTL, 2024. Accessed on October 21, 2024 at: <https://financialservices.wustl.edu/wfin-topic/insurance-risk-management/vehicle-insurance/>.
